# Pathogenic variants of *PROC* gene caused type II activity deficiency in a Chinese family: A case report

**DOI:** 10.1097/MD.0000000000025160

**Published:** 2021-03-26

**Authors:** Hui Zhu, Hongchao Liu, Jingyao Liu

**Affiliations:** Department of Neurology, The First Hospital, Jilin University, Changchun, China.

**Keywords:** Chinese family, hereditary Protein C deficiency, missense mutation, *PROC*, thrombophilia

## Abstract

**Rationale:**

Hereditary Protein C (PC) deficiency is a rare genetic disorder caused by *PROC* gene mutation. In this article, we report a case of PC deficiency in a Chinese family due to a novel *PROC* gene mutation.

**Study Subject:**

The proband presented with recurrent cerebral infarction over the course of the previous 3 years. He was admitted to the hospital due to signs of mental retardation.

**Diagnoses:**

Physical examination, laboratory tests, and magnetic resonance imaging demonstrated that the proband had a manifestation of PC deficiency that included acute cerebral infarction. DNA sequencing analysis revealed a missense variant, c.1015G > A (p.V339 M from valine to methionine) in exon 9 of the *PROC* gene. In addition, Sanger sequencing confirmed that the proband's son was heterozygous for the same variant. Therefore, the *PROC* gene mutation was transmitted in an autosomal dominant inheritance manner.

**Interventions:**

The patient was treated with a daily dosage of Warfarin (3.5 mg) and was scheduled to undergo regular blood coagulation tests.

**Outcomes:**

At the 3-month follow-up appointment, the patient showed improvements in his overall health condition.

**Lessons:**

We identified a novel missense mutation in the *PROC* gene in a Chinese family which caused a decrease in the PC antigen level and recurrent cerebral infarction.

## 1 Introduction

Thrombophilia, a major risk factor for thromboembolic diseases, describes a group of inherited or acquired blood clotting disorders that can be caused by impaired anticoagulation, fibrinolytic proteins, or coagulation factors.^[1]^ The Protein C (PC) anticoagulant pathway is crucial for regulating thrombosis. PC deficiencies are common among Asian patients with inherited thrombophilia.^[2,3]^ Hereditary PC deficiency results from mutations in the *PROC* gene and the severity of symptoms is determined by the presence of homozygous or heterozygous mutations.^[4]^ Homozygous patients present with more severe symptoms including pulmonary embolism, disseminated intravascular coagulation, and deep vein thrombosis in the neonatal period. In contrast, symptoms in heterozygous patients mostly develop in adulthood and these patients often present with deep venous thrombosis.^[5]^

In this report, we present a Chinese family with hereditary PC deficiency caused by a novel *PROC* gene mutation.

## 2 Methods

### 2.1 Subjects

The study protocol was revised and approved by the Ethics Committee of Jilin University, Changchun, China. Patients involved in this study agreed to participate by signing an informed consent form before commencing the study procedures.

### 2.2 Brain imaging

The proband underwent several brain imaging procedures including magnetic resonance imaging and diffusion-weighted imaging (MRI + DWI), susceptibility-weighted imaging (SWI), magnetic resonance venography (MRV), and cerebrovascular digital subtraction angiography.

### 2.3 *PROC gene* mutation analysis

Blood samples were drawn from the proband and the proband's son, and DNA was extracted according to the standard protocols. The DNA was amplified on a GeneAmp Polymerase Chain Reaction system 9700 fast thermal cycler (Applied Biosystems) and sequencing was performed on an ABI 3730XL capillary sequencer (Life Technologies). *PROC* mutation was identified through a comparison of the obtained sequences with a human genome sequence and the pathological mutation was identified using the Human Gene Mutation Database (PMID: 7670104).

## 3 Results

### 3.1 Clinical manifestations

The proband, a 49-year-old male farmer, suffered from recurrent cerebral infarctionx that repeated five times over a 3-year period. Consequently, the patient developed unclear speech and slow reaction and was admitted to the hospital due to signs of mental retardation. The patient had no history of hypertension, diabetes, pulmonary embolism, deep venous thrombosis in the lower extremities, or smoking or drinking problems. However, the patient confirmed that his father and uncle suffered from “cerebral infarctions” repeatedly. Neurological examination revealed sluggish speech and slow reaction time but with normal memory and computational functions as well as normal orientation. Pupil and cranial nerve examinations showed no abnormalities. Sensorimotor functions were normal in all extremities and pathological reflexes were absent.

Laboratory investigations revealed normal findings for the blood counts, blood electrolytes, and liver and renal functions. Similarly, pituitary hormone levels (except gesterol) were within the normal levels. Interestingly, the PC activity (111%, normally 74–100%) was also normal but the PC antigen level was low (<1 μg/dl, normally 1.40–6.30 μg/dl).

**Figure 1:**
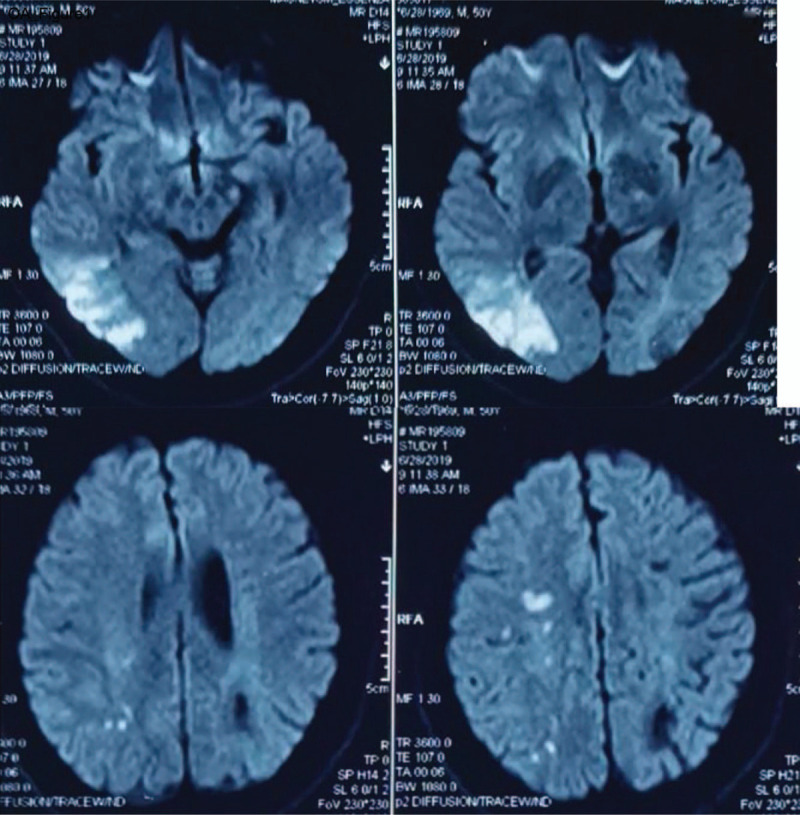
Brain MRI + DWI demonstrating patchy abnormal signals in the right paraventricular, semiovale center and right parietal lobe.

**Figure 2:**
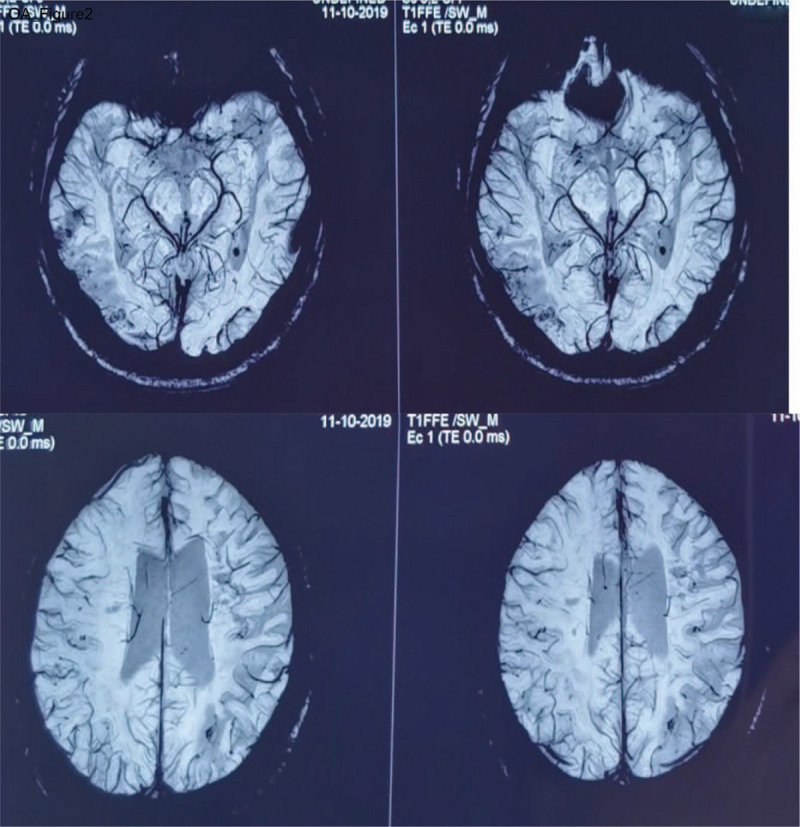
Brain SWI showing abnormal signals in the right temporal occipital lobe and left parietal lobe.

Brain MRI + DWI showed patchy abnormal findings in the right paraventricular, semiovale center and the right parietal lobe (Fig. 1), SWI showed abnormal signals in the right temporal occipital lobe and left parietal lobe (Fig. 2), and the brain MRV and Cerebrovascular digital subtraction angiography were normal. Therefore, the patient was diagnosed with cerebral infarction with PC deficiency.

### 3.2 Genetic analysis

**Figure 3:**
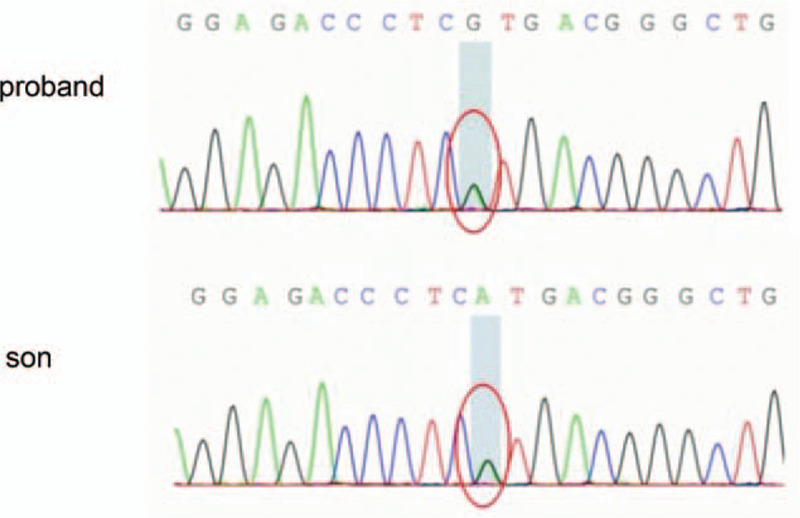
Sanger DNA sequencing profiles. The proband carry a novel mutation c.1015G > A. The proband's son was heterozygous for this mutation.

Genetic analysis revealed a missense mutation G > A (c.1015G > A) that led to an amino acid change (p.V339 M) in exon 9 of the *PROC* gene (Fig. 3). Sanger sequencing confirmed *PROC1* point mutation in the proband and his son. They were both heterozygous for this variant. The proband's father and uncle had died several years earlier and therefore, we could not obtain blood samples.

**Figure 4:**
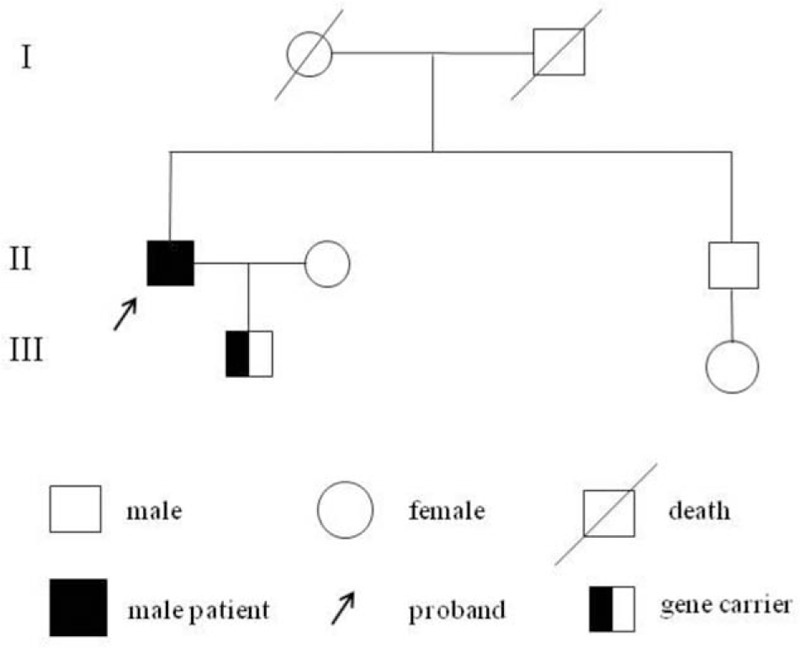
Pedigree of the Chinese family carrying a novel *PROC* mutation.

Based on the clinical manifestations and genotypic analysis results, we constructed the family pedigree (Fig. 4) and our results suggested that *PROC* point mutation led to inherited PC deficiency. The proband was treated with a daily Warfarin regimen (3.5 mg) and underwent regular blood coagulation testing. At the 3-month follow-up appointment, the patient's speech disorder was significantly improved, which positively impacted his quality of life.

## 4 Discussion

Activated PC plays a crucial role in the regulation of coagulation and PC deficiency is often associated with thrombosis.^[6]^ In this study, our results showed a heterozygous mutation of g1015a (v339 → m339) in exon 9 of the *PROC* gene in an adult male patient with recurrent cerebral thrombosis. Interestingly, the patient's PC level was normal, but his antigen level was low. A coagulation test and genetic analysis demonstrated the absence of v339m mutation in the proband's son. The proband's father and uncle had a history of recurrent cerebral infarctions and both of them had died years earlier. Therefore, it was not possible to diagnose whether their thrombosis was caused by the same missense mutation. Nevertheless, there was no family history of venous thrombosis or liver and kidney diseases and the level of PC antigen was normal in other family members. Taken together, we presented a case consistent with type II PC deficiency in this study.^[7]^

Griffin et al demonstrated that PC deficiency is often associated with a family history of recurrent venous thrombosis and low PC antigen.^[8]^ In addition, PC deficiency can also be associated with various clinical manifestations including venous thromboembolism (VTE) and acute life-threatening complications like purpura, fulminant purpura, and disseminated intravascular coagulation as well as severe sepsis.^[9]^ Moreover, activated PC is the main mediator of inflammation.^[10]^ PC is synthesized in hepatocytes and circulates in plasma as a heterodimer complex of heavy and light chains.^[11]^ Activation of PC occurs on the surface of endothelial cells and requires two membrane receptors: endothelial PC receptor and thrombomodulin.^[12]^ Activated PC inactivates activator V and VIII through limited proteolysis, thus inhibiting thrombin production.^[13]^

To date, more than 300 mutations that disturb PC protein synthesis and/or function have been reported. PC deficiency can be classified as quantitative (type I), characterized by reduced levels of PC, or qualitative (type II), in which patients have almost normal antigen levels with reduced activity.^[7]^ In this study, the proband's PC activity was normal, but his PC antigen level was low Therefore, our patient was diagnosed with type II hereditary PC deficiency. The type of PC deficiency can be affected by other factors in addition to the gene mutation.^[6]^ Our results revealed that g1015a mutation did not affect the PC level but decreased the antigen level. Alhenc-Gelas et al previously demonstrated that about 25% of PC type II mutation carriers do not display a phenotype in accordance with the mutation category.^[7]^

In this study, the brain MRI + DWI showed patchy abnormal findings in the right paraventricular, semiovale center and the right parietal lobe. Hereditary PC deficiency can be an independent risk factor for venous thrombosis in the early stage, especially cerebral venous sinus thrombosis (CVST), and increases the risk of CVST recurrence.^[6]^ Systemic lupus erythematosus (SLE) is another risk factor for recurrent thrombosis leading to recurrent venous thrombosis and eventually death.^[14]^ In our study, our proband, his father, and his uncle had recurrent cerebral thrombosis. Their family history indicated the absence of venous thrombosis and the onset age was more than 40 years old.

Heterozygous PC deficiency occurs due to one wild-type and one mutated *PROC* gene. Studies have shown that around 5% of VTE patients may have heterozygous PC deficiency.^[15]^ Moreover, the risk of thrombosis in asymptomatic heterozygous PC deficiency patients increases by 2.5% per year.^[16]^ Therefore, members of our proband's family should be screened and self-monitored for signs of VTE.

Information regarding the impact of *PROC* gene mutation on the clinical phenotype is controversial.^[7,17,18]^ It is likely that the risk of VTE is different among PC mutation carriers. Owing to the fact that hereditary PC deficiency screening is usually based on plasma PC measurements, the heterogeneity of the PC plasma phenotype can lead to an increased risk of misdiagnosis. Therefore, analysis of *PROC* gene mutation can be instrumental in identifying heterozygous individuals. These individuals should be monitored for VTE and physicians should consider administering prophylactic anticoagulant therapy.^[7,19–22]^

Taken together, in this article, we reported a novel *PROC* pathogenic variant p.V339 M associated with the development of *PROC* type II deficiency. Our results can further enhance the available knowledge regarding *PROC* mutations and the resulting phenotypes. Future studies will focus on clarifying the underlying molecular mechanism of p.V339 M mutation.

## Author contributions

Hongchao Liu collected the data. Huizhu analyzed the study results. Methodology and investigation were mainly carried out by Jingyao Lui, respectively. Hui Zhu and Jingyao Liu revised and supervised the whole project. Hui Zhu wrote the manuscript. All authors approved the final version of this manuscript.

**Data curation:** Hongchao Liu.

**Formal analysis:** Hui Zhu.

**Investigation:** Hui Zhu.

**Methodology:** Hongchao Liu.

**Project administration:** Jingyao Liu.

**Writing – original draft:** Hui Zhu.

**Writing – review & editing:** Hongchao Liu, Jingyao Liu.
